# Wearable Sensor for Assessing Gait and Postural Alterations in Patients with Diabetes: A Scoping Review

**DOI:** 10.3390/medicina57111145

**Published:** 2021-10-22

**Authors:** Lorenzo Brognara, Antonio Mazzotti, Alberto Di Martino, Cesare Faldini, Omar Cauli

**Affiliations:** 1 Department of Biomedical and Neuromotor Sciences (DIBINEM), Alma Mater Studiorum University of Bologna, 40123 Bologna, Italy; lorenzo.brognara2@unibo.it (L.B.); antonio.mazzotti@ior.it (A.M.); albertocorrado.dimartino@ior.it (A.D.M.); cesare.faldini@ior.it (C.F.); 2 1st Orthopaedic and Traumatologic Clinic, IRCCS Istituto Ortopedico Rizzoli, Via Giulio Cesare Pupilli 1, 40136 Bologna, Italy; 3 Department of Nursing, University of Valencia, 46010 Valencia, Spain; 4 Frailty and Cognitive Impairment Group (FROG), University of Valencia, 46010 Valencia, Spain

**Keywords:** diabetes, gait, inertial sensor, wearable device, wearable sensor, posture

## Abstract

*Background and Objectives:* Diabetes mellitus is considered a serious public health problem due to its high prevalence and related complications, including gait and posture impairments due to neuropathy and vascular alterations and the subsequent increased risk of falls. The gait of patients with diabetes is characterized by alterations of the main spatiotemporal gait parameters such as gait velocity, cadence, stride time and length, which are also known to worsen with disease course. Wearable sensor systems can be used for gait analysis by providing spatiotemporal parameters and postural control (evaluated from the perspective of body sway), useful for investigating the disease progression. Thanks to their small size and low cost of their components, inertial measurement units (IMUs) are easy to wear and are cheap tools for movement analysis. *Materials and Methods:* The aim of this study is to review articles published in the last 21 years (from 2000 to 2021) concerning the application of wearable sensors to assess spatiotemporal parameters of gait and body postural alterations in patients with diabetes mellitus. Relevant articles were searched in the Medline database using PubMed, Ovid and Cochrane libraries. *Results:* One hundred and four articles were initially identified while searching the scientific literature on this topic. Thirteen were selected and analysed in this review. Wearable motion sensors are useful, noninvasive, low-cost, and objective tools for performing gait and posture analysis in diabetic patients. The IMUs can be worn at the lumber levels, tibias or feet, and different spatiotemporal parameters of movement and static posture can be assessed. *Conclusions:* Future research should focus on standardizing the measurement setup and selecting the most informative spatiotemporal parameters for gait and posture analysis.

## 1. Introduction

Gait impairments such as poor balance, neuropathies and muscle weakness, either together or individually, are common among people with diabetes mellitus (DM), and can lead to gait abnormalities, including incorrect foot pressure distribution. Patients with diabetic polyneuropathy (DPN) also suffer from an altered gait and stability and present a fivefold increased risk of falling or reporting a fall-related injury experienced during standing and walking [1,2,3,4,5].

Patients with diabetes can exhibit slow gait with smaller step lengths and greater step variability compared to healthy individuals, and these gait impairments also affect the patients’ quality of life [6]. Individuals with diabetes and with DPN present smaller step length, reduced duration of single support, higher duration of double support, decreased gait velocity, lower cadence, an increased step-width-to-step-length ratio and greater gait and step variability compared to the control group, probably as a consequence of the range of motion in the lower knee, ankle and first metatarsophalangeal joints and strength in the sagittal plane. Limited dorsiflexion ankle mobility due to a reduced extensibility of the Achilles tendon [7,8] leads to an increased plantarflexion mobility associated with an increase in forefoot pressure [9]. Glycation of collagen in tissue seems to lead to greater tendon stiffness [10,11] which could be a factor enhancing walking impairments [12]. A loss of protective sensation with a repetition of high pressure on the forefoot during the push-off phase of gait may lead to the development of foot ulceration [13]. Sensory impairment can lead to deterioration in the ability of proprioception and can be a key factor for instability of posture [14,15]; in fact, amplification of the postural sway of people with diabetes is associated with peripheral sensory neuropathy [16]. However, DPN may not be the only fundamental cause; visual and vestibular impairments must also be considered [17].

**Figure 1:**
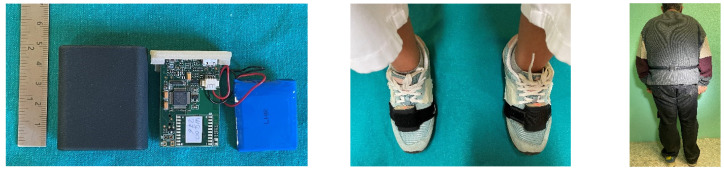
Circuit board of an Inertial Measurement Unit (IMU) and applications.

Gait analysis is usually performed in a laboratory with a set of measurement systems: stereophotogrammetry, force platforms and EMG, but time expenditure and financial constraints limit their use in clinical practice [18], so the availability of effective and reliable tools for gait analysis is paramount. The ability of inertial sensors to measure small movements with a bandwidth and resolution sufficient for the measurement of human movements has made them an excellent alternative for the creation of measurement systems and equipment for medical applications. Inertial sensors have two main advantages. First, their size, weight and energy consumption enable the manufacture of portable systems so small that they reduce or eliminate possible disturbances to natural movement that other, bulkier systems may cause. Second, these devices perform motion measurement with no need for reference points. Unlike optical or magnetic systems, where well-defined spaces with controlled conditions of light and electromagnetic fields are required for their correct operation, systems based on inertial sensors can operate in open spaces. This makes it possible to perform measurements in real environments and not only in laboratories. Thanks to their smaller size and modest costs of their components, inertial measuring units (IMUs) are easy to wear and reachable tools for movement analysis. IMUs consist of an accelerometer, a gyroscope and magnetometer, and can be used to estimate kinematic parameters and the position, the acceleration and the speed produced by the movement with great accuracy. These sensors are provided with an accelerometer that can quantify the acceleration of the human body during motion by measuring the inertia of a mass when subjected to an external force and acceleration. They also contain a gyroscope capable of measuring angular velocities around a predefined axis, and in clinical practice, a 3D system that measures angular velocities in the three orthogonal axes (yaw, pitch and roll) is used as a reference. The accelerometer is often combined with a gyroscope in inertial measurement units (IMU). Figure 1 shows an example of an electronic board with an IMU, a microcontroller, a flash memory for local data storage and a micro-USB port for recharging the battery. However, a wide variety of such methods and tools have been developed for the measurement of human movement in a cheap, fast and as efficient way as possible. Wearable sensor systems can perform gait analysis by providing spatiotemporal parameters useful for investigating the progression of gait problems in patients with various neurological problems such as diabetic neuropathy, without the need for a specialized laboratory for motion analysis, and measurements can be performed even in the domestic context of the patients [19,20,21,22].

This scoping review aims to summarize the scientific evidence on the usefulness of IMUS for the measurement of gait and postural problems in patients with diabetes. In addition, special interest has been given to the experimental details of the different protocols and measurement methods used in the scientific literature such as the number and anatomical position of the sensors and the analysis of the different spatiotemporal or postural parameters analysed. We focus on inertial sensors for gait and posture analysis both outdoors or at home and in the clinical setting, to evaluate the possible application of these wearable sensors in the analysis of movement during tasks related to activities of daily living.

## 2. Methods

### Review Process: Search Strategy and Selection Criteria

In May 2021, a professional librarian performed an electronic search in several databases, namely PubMed (Medline since 1950), Ovid (Biosis, Cinahl), Cochrane (Central, Dare/CRD, HTA) covering dates of publication between 2000 and May 2021. The search strategy included the following keywords: gait; postural sway; gait disorder; walking; kinematic; gait analysis system; wearable sensor; accelerometery; inertial sensor; wearable device; gait analysis device; diabetic neuropathy; diabetes mellitus not amputation. The language was restricted to English. The articles reviewed did not focus on treatment approaches, but instead on the evaluation of gait and posture of patients with diabetes, using wearable sensors.

A scoping review was performed to assess spatiotemporal parameter variables of gait in patients with diabetes mellitus using wearable sensors.

An electronic search was performed first in PubMed on 1 March 2021, using the following search string:

(diabetes * [Title/Abstract]) AND (accelerat * [Title/Abstract] OR acceleromet * [Title/Abstract] OR inertia * [Title/Abstract] OR gyroscop * [Title/Abstract] OR “wearable sensor *” [Title/Abstract] OR “body-fixed sensor *” [Title/Abstract]) AND (gait * [Title/Abstract] OR walk * [Title/Abstract]); (“diabete” [All Fields] OR “diabetes mellitus” [MeSH Terms] OR (“diabetes” [All Fields] AND “mellitus” [All Fields]) OR “diabetes mellitus” [All Fields] OR “diabetes” [All Fields] OR “diabetes insipidus” [MeSH Terms] OR (“diabetes” [All Fields] AND “insipidus” [All Fields]) OR “diabetes insipidus” [All Fields] OR “diabetic” [All Fields] OR “diabetics” [All Fields] OR “diabets” [All Fields]) AND (“postural” [All Fields] OR “posturally” [All Fields] OR “posture” [MeSH Terms] OR “posture” [All Fields] OR “postures” [All Fields] OR “postured” [All Fields] OR “posturing” [All Fields]) AND (“sensor” [All Fields] OR “sensor s” [All Fields] OR “sensoric” [All Fields] OR “sensorics” [All Fields] OR “sensoring” [All Fields] OR “sensorization” [All Fields] OR “sensorized” [All Fields] OR “sensors” [All Fields]).We performed the searches with other databases in other to find articles not included in Pubmed searches.

**Figure 2:**
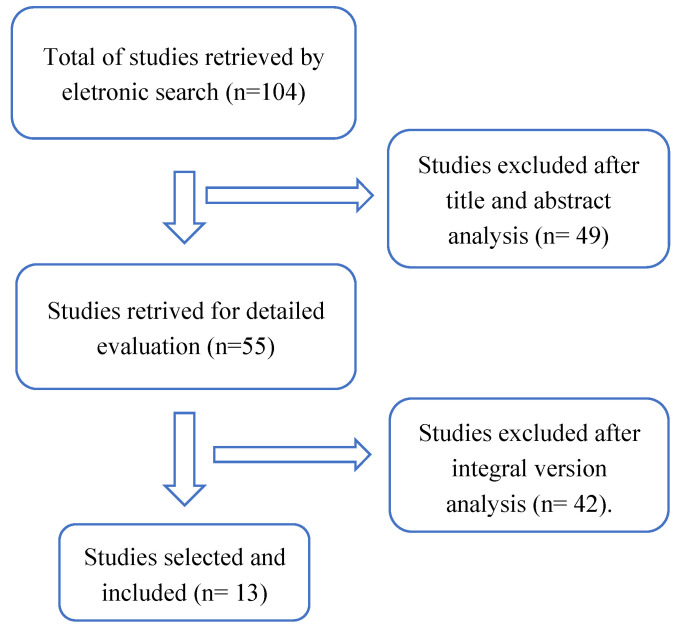
Flow diagram of article selection process through the scoping review.

We excluded conference proceedings, articles reporting only results from kinetic and kinematic joint-angle variables, studies that did not assess gait or posture with only wearable sensors (no gait analysis in laboratory or with a balance/force platform or baropodometric and stabilometric platforms) and those that assessed gait over a walking distance shorter than five meters. Figure 2 presents a flowchart of the review process.

## 3. Results

The main characteristics of the studies analysed in the scoping review are summarized in Table 1 and Table 2. We present the most commonly analysed parameters to detect gait and postural disturbances. Regarding the anatomical location of the IMUs sensors we identified ankles, tibias (shanks), feet and lower back.

### 3.1. Characteristics of Patients

The studies analysed included a minimum of 12 and a maximum of 151 diabetic patients. Most of the studies analysed (two thirds) specified the presence of DPN. However, the remaining studies did not specify the presence of DPN, which makes it difficult to compare the alterations in the spatiotemporal parameters of gait between the different studies.

### 3.2. Sensor Number and Placement

Various numbers of wearable sensors were used and placed on different parts of body, as shown in Table 1 and Table 2.

The published studies use an average number of three sensors in their measurement protocols, but it is possible to use only one sensor (on the back) but undoubtedly the most used configuration is the one that uses measurements with five sensors [15,20,21,22,23]. When a single sensor is used, it is most often placed at the level of the L5 lumbar vertebra. The lumbar position (alone or with other sensors) has in fact been used in seven of the studies analysed in the review [15,17,19,20,21,22,23]. Other fairly common positions for gait measurement are the location of IMUs on the ankles (or in some cases on both tibias). In two of the studies [16,18] simultaneous positioning on both ankles, tibias and feet was not useful and sometimes gave less-reproducible results. 

Due to the variety of combinations and anatomical locations, a clear indication of the minimum number of sensors needed to obtain a good measurement of gait and posture cannot be given. The accuracy and comfort for the patient during basic activities of daily living in relation to the different possible IMU positions deserves to be analysed in future studies.

### 3.3. Spatiotemporal Parameters

Studies analysing human motion with IMUs and studies estimating the risk of falls in neurologically impaired patients have identified different parameters for a proper analysis of motion through inertial sensors: (1) the gait onset velocity, (2) the mean stride velocity during steady state gait, (3) the coefficient of variation of stride velocity during gait (gait stability), (4) the mean amplitude of body centre of mass movement during the performance of each stride in the mediolateral direction, (5) the mean amplitude of body centre of mass movement during each stride in the anteroposterior direction, (6) the average plantar double stance phase expressed as a percentage of stride time, (7) the average time taken to perform the strides, (8) the average stride length, and (9) the number of steps required to reach a steady state of gait which is defined as the ability to control the centre of mass relative to the base of support when in predictable and unchanging conditions, such as when sitting or standing [35]. Wearable inertial devices have recently been used to assess spatiotemporal parameters of gait in everyday life situations. People with diabetes often have an abnormal gait, which probably contributes to elevated plantar pressures and risk of foot ulcers and risk of falls.

Significant differences were found among the selected articles for the spatiotemporal parameters of gait, as shown in Table 1. This is clearly related to the different configurations (the number and positions of the sensors that were used). As expected, gait speed in the diabetic patient is the most commonly studied parameter (in fact it has been analysed in all the articles reviewed). Other studies have evidenced gait disturbances in diabetic patients by measuring stride velocity, which should reflect the same underlying parameter. Gait cadence, which is also reported as step frequency, was analysed in 55% of the studies. Stride length, which can be especially useful in assessing patients with diabetes (who often have short strides) was also analysed in studies that also measured cadence. Steady-state gait, also referred to as “steady-state” activity, in which the body neither accelerates nor decelerates, is useful for assessing the presence of DPN and was the least studied (33% of studies) and merits evaluation as a measure for assessing fall risk in patients with diabetic neuropathy. In some studies (see Table 1), in addition to gait characteristics, stride characteristics are also often evaluated. The variability of stride and gait characteristics is lower than the normal value in patients with diabetes. Among the variabilities, stride-time variability was only studied in one article [31] and therefore deserves further attention in future studies.

### 3.4. Differences in Gait Analysis and Spatiotemporal Parameters between Patients with Diabetes (with or without DPN) and Healthy Individuals

Some authors have shown that patients with DPN have a slower gait velocity than healthy individual subjects [36,37], but the literature presents a high level of heterogeneity on this topic; in fact other authors [8,38] reported that DPN participants walked faster than both healthy people and patients with diabetes without DPN.

Meta-analysis results combining data from studies for walking speed and stride length between the patients with diabetes (with or without DPN) and healthy individuals demonstrated no significant difference in walking speed and stride length. However, there is good evidence to state that DPN patients had a longer percentage duration in the stance phase of gait [39].

Particular attention must be paid to some comorbidities such as cognitive and attention impairments, because a slower gait velocity, shorter strides and reduced double-support time with an increased gait variability have been associated with patients with diabetes and brain dysfunction, leading to a high risk of consequent falls [40].

### 3.5. Protocol and Analysis of Posture Parameters

In all the studies reviewed, postural assessment has been analysed in patients with diabetes by measuring the motion of sway of the body during standing with feet close together, standing still or after visual perturbation (eyes-open or closed) or somatosensory perception disturbances (firm/foam surfaces). Additionally, in terms of postural parameters analysed, there is uniformity in parameters such as total sway area (cm^2^), medial–lateral sway (cm) and anterior–posterior sway (cm) considered by all authors. Significant differences were found in the number and positions of IMUs used. The most used setup is with two sensors [32,33] but we find a wide variety of combinations; regarding the number and position of the sensors a consensus among the clinical research community has yet to be achieved.

### 3.6. Comparisons of Gait and Postural Alterations in Diabetic Patients Using Wearable Sensors and Other Methods which Assess Motor and Sensitive Alterations

Caron et al. [25] evaluated the association between alterations in gait and the rates of oxygen consumption during walking in patients with type II diabetes by using a breath-by-breath gas analyser. The metabolic rates when walking were significantly higher for diabetic patients than for healthy subjects and it was significantly associated with higher step frequency. Decreasing step length by increasing step frequency may be the result of an adaptation made by these patients in order to increase perceived stability when walking. However, these adaptations could increase the internal work needed to move the lower limbs and thus may help explain the higher cost of walking observed among T2D patients [41]. De Bruin et al. [30] evaluated the outcomes of the gait analysis with other diagnostic tests, such as the neurometer device, to measure the sensory-nerve conduction threshold by means of current perception threshold levels to diagnose and quantify hyperaesthesia in patients with DPN. The Rydel-Seiffer tuning fork test was used to assess the vibratory threshold perception at the base of the great toe, and is a good predictor for impairment of the vibratory senses, and therefore, is also usable to diagnose neuropathy. The third test used was the Semmes–Weinstein monofilament test, a good test to diagnose, but not to quantify, neuropathy. All these measurements correlate with neuropathy severity assessed by IMUs [30]. Najafi et al. [30] found an excellent correlation was observed between the area of sway of the centre of mass measured by the sensors and the area of pressure sway measured by a pressure platform. Toosizadeh et al. [33] evaluated DPN by using the American Diabetes Association criteria based on insensitivity to a 10 g Semmes–Weinstein monofilament. Additionally, vibration perception threshold was recorded to quantify the level of neuropathy with a cutoff of 25 mV as an indicator of neuropathy at recommended plantar foot sites. Both measures correlate with postural alterations recorded by IMUs technology. The improvement in gait and postural alterations in diabetic patients with DPN after plantar electrical stimulation observed with IMUs [29] was not correlated with a similar improvement measured through the mobility tiredness scale, suggesting the changes induced by this intervention were likely to be small, or that IMUS analysis was more sensitive than the classical tools based on self-reported questionnaires.

## 4. Discussion

Both nervous and vascular alterations are significant long-term complications in patients with diabetes, and they account for significant morbidity and mortality and the risk of falls, so the evaluation of gait and posture in patients with DPN and vascular problems in feet, leading to alterations in biomechanical spatiotemporal variables of gait, is clinically important to diagnose and tailor interventions to reduce the adverse outcomes [42,43,44,45,46]. Meta-analysis results suggest that DPN patients expended a longer period of time in the stance phase compared to patients with diabetes without DPN and healthy individuals [39]. Postural stability is a strong predictor of falls, and consequently the assessment of postural parameters are essential [47]. Their low cost and lightweight, portable nature makes inertial sensors an effective tool to be implemented into a diabetic clinic.

Biomechanical investigation using wearable inertial sensors allows the identification of gait abnormalities that also may adversely affect foot ulceration. In fact, people with foot ulcers had a significantly slower walking speed and smaller step length compared with healthy control individuals [48]. In summary, one of the most important spatiotemporal parameters that seems significantly different in DPN patients compared to patients with diabetes without DPN and healthy individuals is longer stance time. This is coupled with elevated plantar pressure in DPN patients, contributing to a susceptibility to skin damage and ulceration [39].

Inertial sensors are microelectromechanical systems including accelerometers and gyroscopes that can be worn by the patient without restrictions for several hours of measurements [49], and they represent a unique tool for analysing gait impairment in natural environments. Thus, they are useful for implementing strategies to reduce the risk of falls which represents a major public health problem [50]. Recent reports suggest that IMUs, besides being useful in evaluating and to monitoring gait alterations in patients with neurodegenerative diseases such as Parkinson’s disease [51,52,53], are useful for; patients with osteoarthritis [54], with benign paroxysmal positional vertigo, the most common peripheral vestibular disorder, leading to balance difficulties and increased fall risks [55] and walking disturbances in sarcopenic patients [56], they are also useful for gait event detection and analysis of gait alterations in patients with diabetes secondary to DPN [23,24,27,28,29,31]. In spatiotemporal gait parameters recorded using a wearable sensor in patients with DPN, Kang et al. showed that gait initiation steps and dynamic balance may be more sensitive than gait speed for detecting gait deterioration due to DPN [23], and Najafi et al. demonstrated that gait alteration in patients with DPN is most pronounced while walking barefoot over longer distances [31]. Spatiotemporal gait parameters are detected according to the specific features appearing on the accelerometer and gyroscope signals, but the heterogeneity of gait patterns, sensor placement and analysis algorithm may influence the validity of the results.

In addition to the reported and structured analysis based on Table 1 and Table 2, we also observed further promising approaches for assessing patients with diabetes using wearable IMUs which could be relevant for further investigation. Indeed, the presence of cognitive impairment with DPN increased gait variability only for the dual-task walking condition, and it exacerbates the risk of falls in diabetic patients [24].

First, in addition to the most commonly used spatiotemporal parameters to assess gait, we also identified less commonly used parameters whose interest seems relevant for patients with DPN, such as the variability of stride and stride-time variability.

Regarding the type of tests used to assess gait with inertial sensors, it is advantageous to use gait tests with longer distances, e.g., more than 5 m, to obtain a more homogenous gait pattern, e.g., less affected by the start and stop phases of gait which lead to a change in the spatiotemporal parameters thus leading to higher variability in parameters’ values. In fact, a variability in the distance walked was observed in the studies analysed in the type of gait test, which may be an important aspect for future research on the standardization of this parameter. In fact, De Bruin et al. demonstrated that the reliability of gait speed, cadence, stride length and step length measurements on different surfaces and in dual-task conditions performed outdoors was high, with excellent results in long test distances [30].

Finally, all the results suggested the feasibility of using wearable platforms to quantify motor performance and balance during a clinical visit. Some studies present values from healthy controls, which can help in evaluating the usefulness of parameters in characterizing gait impairments in patients with diabetes compared to patients who are of similar age and are sex-matched, without diabetes. Comparisons between DPN and control groups showed differences for all spatiotemporal and control variables except for stride length [27], and DPN worsens gait function. Diabetic foot ulcers magnify the deterioration beyond DPN [28,57,58]. Consensus on the assessment protocols and parameters is still lacking [59] and future research is needed in order to compare measurements by IMUs with gold standards (or methods that were previously compared with gold standard technologies). This may also impact the reliability of and comparison between the different results obtained to date, and the future development and research into IMUs for clinical applications should address these issues. Tailored sensor-based interactive exercise with real-time feedback is useful for improving postural stability during daily physical activities and is another promising field of application. This was not covered in this review but certainly will be subject of future research studies [60,61]. This scoping review presents some limitations that could be accounted for in future studies. First, the low number of studies (especially for posture analysis) with different experimental designs published on IMUs’ use in DPN did not allow for suggestion of the best experimental protocol to measure DPN in future studies. Second, we have not looked in the databases for any scientific reports written in other languages besides English and Spanish, thus we cannot exclude that other studies with IMUs could have been published. The information about the sensitivity and specificity of IMUs to detect DPN as well as the effects of therapeutic interventions to improve glycaemic control, and thus DPN, have not been studied so far. The only study comparing IMUs data on gait between DPN and control groups found the greatest discriminatory power with good discriminatory power (area under the curve >0.8) for the parameters of walking speed and step time [27].

In comparison with other motion measurement devices, wearable sensors and IMUs have the advantage of being lightweight and portable, which means the subjects can move relatively freely, making them ideal for understanding gait changes in patients with diabetes after taking measurements in real-life conditions. In fact, during activities of daily life, patients need to move across challenging surfaces and irregular terrain that has been shown to have a negative impact on gait parameters and the risk of falling [62,63,64] in patients with DPN or with high levels of HbA1c that can lead to diabetic peripheral neuropathy [65,66]. The effects of age on gait and postural alterations in diabetic patients recorded by IMUs was analysed only in two studies. Zhou et al. [26] found a significant correlation between age and gait performances among people with diabetes for stride velocity and double support, being stronger with diabetic patients with terminal renal diseases undergoing haemodialysis. In contrast, D’Silva et al. [34] found no differences in any measures of postural sway in people with type 2 diabetes compared to controls. Previous studies using centre of pressure displacement calculated from force platforms have shown that people with type 2 diabetes, particularly those with DPN, have greater range and velocity of sway compared to age-matched controls, when standing on a firm surface with and without visual input [67]. This difference may be because the subjects enrolled in this study were younger (mean age 52 years old) and with quite well controlled diabetes (average HbA1c 7.8%). Future studies examining glycaemic control, duration of diabetes, type of diabetes, sex, severity of neuropathy, and their relationship to postural and gait alterations are necessary to identify subjects with high fall risk.

The spatiotemporal variables recorded by the IMUs warrant further studies in terms of their applications in detecting gait and posture impairments in diabetic patients in clinical settings and at home. Therefore, the choice of the number of sensors and sensor locations should be based on the clinical relevance and required accuracy of the specific gait parameters, as well as the ease of use of the setup. Gait velocity, cadence and stride length were the most frequently recorded parameters, and further work should be conducted to choose the most sensitive ones for different outcomes, such as for fall risk assessment and efficacy of treatment to improve the diabetic foot, to assess the most representative and discriminating gait parameters between the gait of people with and without diabetes.

## Figures and Tables

**Table 1:** Characteristics of wearable sensors and spatiotemporal parameters. X: item assessed in the study.

Patients, IMU Location and Spatiotemporal Parameters Analysed	Kang et al. (2020) [23]	Kang et al. (2020) [24]	Caron et al. (2018) [25]	Zhou et al. (2018) [26]	Esser et al. (2018) [27]	Ling et al. (2020) [28]	Najafi et al. (2017) [29]	De Bruin et al. (2012) [30]	Najafi et al. (2013) [31]
Number of diabetes subjects	38	44	20	151	17	39	28	31	12
Mean age	72.6	66.5	57.5	78.0	63.0	64.3	56.0	Not specified (participants had between 50–70 years old)	60.0
Diabetes history, years	Not reported	Not reported	10.6	Not reported	24 ± 16	Not reported	Not reported	Not reported	10 ± 13
Assessment of DPN	X	X			X	X	X		X
2 IMUs on both ankles or on both tibias (shanks)	X	X		X		X	X		X
IMUs on both feet									
IMU on lower back	X		X		X	X	X	X	X
Other anatomical locations (#IMUs)	2 on thighs					2 on thighs	2 on thighs		2 on thighs
Number of IMUs used for the measurements	5	2	1	2	1	5	5	1	5
Gait speed (stride velocity)	X	X	X	X	X	X	X	X	X
Cadence (or step frequency)	X		X		X		X	X	
Stride length		X	X	x	X	X	X		
Stride length variability		X							X
Stride time (Gait cycle time)		X					X		
Steady-state gait	X					X			X
Step length								X	
Step time					X			X	
Step time variability									X
Double support (time or %)				X		X			
Domicile	X								
Distance covered	12 m	12 m	200 m	15 m	10 m		10 m	31 m	20 m
Outdoor			X					X	
Clinical environment		X		X	X		X		X

**Table 2:** Characteristics of wearable sensors and postural parameters. X: item assessed in the study.

Patients, IMU Location and Postural Parameters Analysed	Najafi et al. (2010) [32]	Toosizadeh et al. (2015) [33]	D’Silva et al. (2017) [34]	Najafi et al. (2017) [29]
# DM subjects	17	18	52	28
Mean age	59.2	65.0	Not specified (participants had between 40–65 years old)	56.0
Diabetes history, years	Not reported	19 ± 11	Not reported	not reported
DPN	X	X		X
2 IMUs on both ankles or on both tibias (shanks)	X (shin)	X (shin)		X
IMUs on both feet				
IMU on lower back	X	X	X	X
Other locations (#IMUs)				2 on thighs
# IMUs	2	2	1	5
firm/foam surfaces	X		X	
Eyes closed	X	X	X	X
Eyes open	X	X	X	X
30 seconds with feet close together	X		X	X
15 seconds with feet close together		X		
Hip sway (deg^2^)	X	X		X
Ankle sway (deg^2^)	X	X		X
Total sway area (cm^2^)	X	X	X	X
Medial–lateral sway (cm)	X	X	X	X
Anterior–posterior sway (cm)	X	X	X	X
Range (cm/s^2^) of acceleration, in AP and ML directions			X	
peak velocity (cm/s) in AP and ML			X	
Domicile				
Clinical environment	X	X		X

## Data Availability

Not applicable.
